# Identification of a limb enhancer that is removed by pathogenic deletions downstream of the *SHOX* gene

**DOI:** 10.1038/s41598-018-32565-1

**Published:** 2018-09-24

**Authors:** Isabella Skuplik, Sara Benito-Sanz, Jessica M. Rosin, Brent E. Bobick, Karen E. Heath, John Cobb

**Affiliations:** 1 Department of Biological Sciences, University of Calgary, 2500 University Drive N.W., Calgary, Alberta T2N 1N4 Canada; 2 Instituto de Genética Médica y Molecular (INGEMM), IdiPAZ and Skeletal dysplasia multidisciplinary unit (UMDE), Hospital Universitario La Paz, Universidad Autónoma de Madrid, P° Castellana 261, 28046 Madrid, Spain; 3 CIBERER, ISCIII, Madrid, Spain

## Abstract

Haploinsufficiency of the human *SHOX* gene causes Léri-Weill dyschondrosteosis (LWD), characterized by shortening of the middle segments of the limbs and Madelung deformity of the wrist. As many as 35% of LWD cases are caused by deletions of non-coding sequences downstream of *SHOX* that presumably remove an enhancer or enhancers necessary for *SHOX* expression in developing limbs. We searched for these active sequences using a transgenic mouse assay and identified a 563 basepair (bp) enhancer with specific activity in the limb regions where *SHOX* functions. This enhancer has previously escaped notice because of its poor evolutionary conservation, although it does contain 100 bp that are conserved in non-rodent mammals. A primary cell luciferase assay confirmed the enhancer activity of the conserved core sequence and demonstrated that putative HOX binding sites are required for its activity. This enhancer is removed in most non-coding deletions that cause LWD. However, we did not identify any likely pathogenic variants of the enhancer in a screen of 124 LWD individuals for whom no causative mutation had been found, suggesting that only larger deletions in the region commonly cause LWD. We hypothesize that loss of this enhancer contributes to the pathogenicity of deletions downstream of *SHOX*.

## Introduction

In addition to disturbing coding sequences, chromosomal aberrations can cause genetic disease when they remove, modify or displace *cis*-regulatory elements necessary for the control of gene expression^1^. Within this context, the enhancers that regulate tetrapod limb development are among the best studied^2^, including the zone of polarizing activity regulatory sequence (ZRS) that controls sonic hedgehog (*Shh*) expression in developing limbs^3^. The ZRS is uniquely required for the expression of *Shh* for anterior-posterior patterning of the distal portion of the limbs. Several point mutations cause a gain-of-function of the ZRS resulting in polydactyly in many species including humans^3^. While the discrete ZRS has served as a prototype for understanding the function of enhancers during limb development, in many cases numerous enhancers dispersed over large genomic regions control the expression of genes active in limb patterning^4,5^. For example, the *HoxD* genes that pattern the distal limb fit this dispersed model of regulation^6^. Similarly, multiple enhancers control the expression of *Gdf5* in the developing joints of the limbs^7^. Overall, genes coding for transcription factors important during limb development have been estimated to have a median number of eight enhancers regulating their expression, indicating that genes controlled by a single discrete enhancer might be exceptions to an otherwise general rule^5^. Animal models have been crucial for our understanding of how mutations and chromosomal rearrangements result in limb deformities in humans, helping to define which genes are regulated in a discrete or dispersed fashion^8^.

**Figure 1:**
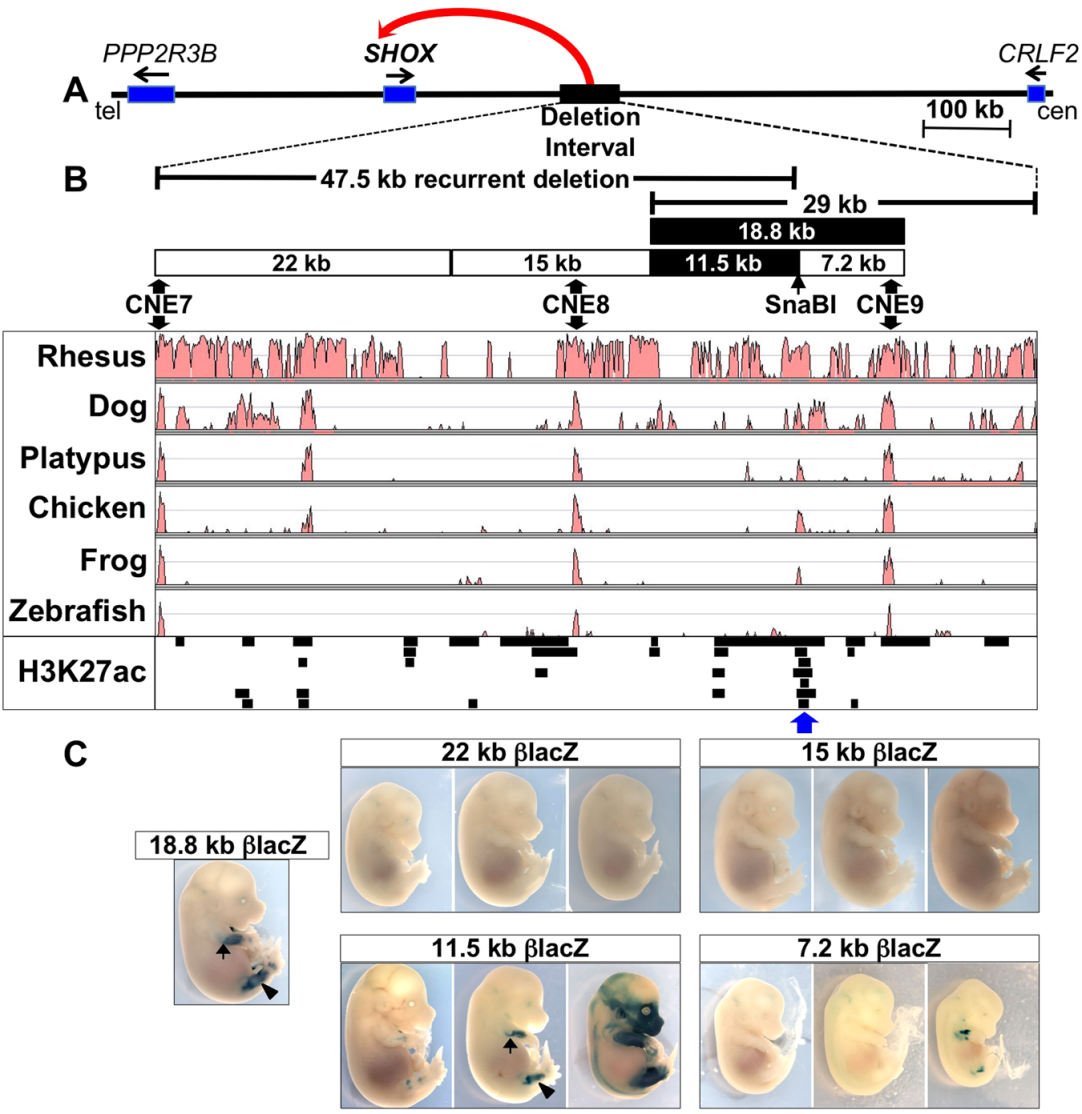
ZED activity is located within an 11.5 kb sequence that does not include CNE9. (**A**) Schematic of ~1 Mb of the human X and Y chromosomes at the *SHOX* locus with a red arrow indicating the location of a presumed enhancer or enhancers within an interval frequently deleted in LWD and ISS patients. (tel = telomeric, cen = centromeric, blue rectangles indicate genes) (**B**) Expanded view of the 65.7 kb deletion interval extending from the telomeric breakpoint of the ~47.5 kb recurrent deletion^17^ to the centromeric boundary of the 29 kb smallest region of overlapping (SRO) deletion from Benito-Sanz *et al*.^9^. The rectangles indicate the fragments tested in the transgenic assay, which are aligned with the deletion intervals and with a plot of the evolutionary conservation of the region (below), as generated with VISTA for the indicated species^63^. The fragments found to have limb enhancer activity are in black; white rectangles indicate fragments that did not have reproducible limb activity. The H3K27ac track shows the location of regions enriched for H3K27 acetylation in human embryonic limbs at E33, E41, E44, E47 (two replicates per stage, except for E41 for which only one replicate showed enrichment in this region) as reported by Cotney *et al*.^29^. Each row of this track represents a different sample arranged from E33, replicate 1 at the top, to E47 replicate 2 at the bottom. The blue arrow indicates the only region with H3K27ac in all of these samples. (**C**) Sets of three E13.5/E14.5 embryos carrying the indicated transgenes; each embryo represents an independent genomic insertion. Only the 11.5 kb βlacZ transgene has reproducible limb staining, which appears similar to that of the previously reported 18.8 kb transgene staining as shown in the embryo at left (in two representative embryos, the arrow points to staining in the forelimbs and the arrowhead indicates hindlimb staining).

The *cis*-regulatory control of the human short stature homeobox gene (*SHOX*) is an emerging paradigm in this field^9,10^. Deletions of non-coding sequences that overlap in an interval approximately 250 kb downstream of *SHOX* on the pseudoautosomal region 1 (PAR1) of the X and Y chromosomes cause a large number of Léri-Weill dyschondrosteosis (LWD, MIM 127300) cases, as many as 35% in some cohorts and a small proportion of cases with Langer mesomelic dysplasia (LMD, MIM 249700) and idiopathic short stature (ISS, MIM 300582)(Fig. 1A)^9,11–13^. Presumably these deletions remove an enhancer or enhancers necessary for *SHOX* expression in developing limbs resulting in *SHOX* haploinsufficiency, although the precise location of these elements has not been determined.

Individuals with either downstream deletions or disruptions of *SHOX* coding sequences have a similar shortening of the middle (zeugopodal) limb elements^9^. LWD is a dominantly inherited skeletal dysplasia that is characterized by disproportionate short stature, mesomelic limb shortening, and Madelung deformity of the forearm: the bowing of the radius, distal dislocation of the ulna and triangulation of the carpal bones^14^. A more severe skeletal dysplasia (LMD) is caused by homozygous or compound heterozygous mutations of *SHOX* or its enhancer regions. LMD is characterized by severe disproportionate short stature due to both mesomelic and rhizomelic shortening of the limbs. ISS is classified as individuals with a height below -2 SDS in the absence of known specific causative disorders.

Others have used evolutionary sequence conservation as a guide to locate potential enhancers downstream of the *SHOX* gene. In this way, an Evolutionarily Conserved Sequence designated ECS4 was identified as a limb enhancer candidate within the critical deletion interval downstream of *SHOX*^15^. ECS4, now commonly known as CNE9 (Conserved Non-coding Element 9), was shown to activate transcription from an episomal reporter construct when electroporated into chick limb buds and in a luciferase assay in U2OS cells^15,16^. However, in these experiments enhancer activity was only tested in the specific cells that were electroporated. Therefore, it was not clear whether the enhancer was tissue-specific since reporter activity was not tested in all tissues of a developing embryo from a construct integrated into the genome. Furthermore, many of the deletions identified in individuals with LWD and ISS do not include CNE9^17,18^ indicating that the limb-specific enhancer(s) may lie elsewhere. Most notably, an identical, recurrent 47,543 base pair (bp) deletion (abbreviated 47.5 kb) identified by array CGH and breakpoint PCR sequencing appears to be a major cause of LWD (location shown in Fig. 1B)^17^. Since this deletion does not remove CNE9, the pathogenic mechanism was thought to be due to the loss of another conserved sequence called CNE7 (also known as ECR1), although the limb-specific enhancer activity of this sequence was not demonstrated^17^. The identical 47.5 kb deletion was found in 15.3% (19/124) and 12.9% (17/132) of LWD patients in two separate cohorts^17,18^. Another study found that 19 of 28 LWD patients with non-coding deletions downstream of *SHOX* carried the 47.5 kb deletion^19^. The penetrance of the LWD phenotype is variable in people carrying the 47.5 kb deletion, including a few that do not have short stature^19^. However, it is unclear if the variability is any greater than that of patients with disruptions of the *SHOX* coding sequence, which also show variable penetrance^19,20^.

Candidate enhancer sequences from the human genome have been analyzed extensively using transgenic mouse assays^21^. This is complicated in the case of *SHOX*, since mice, the most common model for such studies, lack a *SHOX* ortholog. However, since the fundamental gene regulatory networks controlling limb development are common to all tetrapods^22,23^, we predicted that *SHOX* regulatory elements would be faithfully active in a mouse transgenic model. Many studies support such an approach. For example, human-specific enhancer activities have been revealed in transgenic mice^24^ and a recent study showed that the ZRS of the coelacanth fish has similar activity and can functionally substitute for the mouse ZRS despite an evolutionary separation of approximately 400 million years^25^. In these studies, candidate sequences are cloned upstream of a minimal promoter and a reporter gene (usually lacZ). Embryos carrying the reporter transgene constructs are produced by pronuclear injection of one-cell mouse embryos using standard techniques. Although these constructs are incorporated randomly into the genome, position effects can be distinguished from specific and reproducible patterns by collecting multiple transgenic embryos, with each representing an independent genomic insertion. Typically, an enhancer is scored as having a specific activity when three embryos display a similar pattern of expression^21^. Such studies have proven to be an effective method to identify the precise sequences whose modification or removal cause human disease. For example, the ZRS was identified by this technique^3^. Therefore, we have used a similar strategy to search for the enhancer(s) controlling *SHOX* limb expression.

Previously, we used a transgenic mouse assay to show that an 18,796 bp (abbreviated 18.8 kb) genomic fragment containing CNE9 has enhancer activity in developing limbs, specifically in the zeugopodal region where *SHOX* function is critical (genomic location and activity is shown in Fig. 1B,C)^26^. This result supported the validity of using a mouse transgenic model to study *SHOX* enhancers. We also found that a reporter transgene containing only CNE9 did not have reproducible limb expression, suggesting that the limb-specific activity lies elsewhere within the 18.8 kb fragment^26^. We will refer to the activity within the 18.8 kb transgene as the ZED, or zeugopodal enhancer downstream of *SHOX*. Here we report that ZED activity is located within a previously overlooked 563 bp fragment that contains sequences conserved in non-rodent mammals, but not in other vertebrates. We also demonstrate that putative HOX binding sites are required for the enhancer’s activity *in vitro*.

## Results

### The ZED is found within an 11.5 kb genomic fragment that does not contain CNE9

We sought to find the precise genomic location of the ZED and to determine if it is removed by the 47.5 kb recurrent deletion (Fig. 1B). We first used a transgenic mouse assay to screen 11.5 and 7.2 kb sub-fragments of the 18.8 kb transgene previously shown to contain ZED activity^26^. The subdivision of the 18.8 kb fragment was guided by the breakpoint of the recurrent deletion (genomic coordinate ChrX:828,092 (GRCh37/hg19)). For cloning, we used a SnaB1 restriction site located 426 bp centromeric to the deletion breakpoint, so that all sequences of the 18.8 kb fragment that are removed by the recurrent deletion are found in the 11.5 kb fragment (Fig. 1B). In order to determine whether there were other limb enhancers within the 47.5 kb deletion, we also used the transgenic assay to test the enhancer activity of 22 kb and 15 kb fragments that spanned sequences of the 47.5 kb deletion that are not within the 11.5 kb fragment (Fig. 1B). In each case, the assay was performed by cloning the genomic fragments upstream of a human β-globin minimal promoter adjacent to lacZ (βlacZ) as previously described^26^. The resulting constructs were injected into pronuclei of one-cell stage embryos and transient transgenic embryos were isolated 13 or 14 days later^26^. In one case, permanent lines were created (the 7.2 kb βlacZ transgene). Embryos of this developmental range were chosen because of the previously determined timing of the 18.8 kb βlacZ transgene activity^26^ and because *SHOX* is expressed at a comparable stage in human fetuses^27^. Furthermore, *SHOX* mutations are known to cause shortened, malformed limbs as early as 12 weeks gestation, indicating an early function for *SHOX* in developing limbs^28^.

In total, we used the transgenic assay to screen 55 kb of the human genome for enhancer activity, including all of the sequences removed in the recurrent 47.5 kb deletion, plus 7.2 kb extending further centromeric to and including CNE9 (Fig. 1B). Three representative E13.5/E14.5 transgenic embryos for each fragment are shown in Fig. 1C with the complete results summarized in Table 1. Among the transgenes tested, only the 11.5 kb βlacZ transgene showed reproducible limb enhancer activity with staining similar to that of 18.8 kb βlacZ (Fig. 1C). Although one of six lines carrying the 7.2 kb βlacZ transgene (at right, Fig. 1C) showed limb staining, this was judged to be a position effect since its activity was distinct from that of the ZED (see Supplementary Fig. S1) and it was present in only that single line. Therefore, the 11.5 kb fragment contains the ZED and this is the only limb enhancer detected within the 55 kb as tested in our E13.5/E14.5 transgenic embryo assay. Notably, the 11.5 kb ZED fragment does not contain any of the highly conserved sequences previously suspected to function as *SHOX* limb enhancers (CNE7, CNE8 or CNE9) (Fig. 1B)^16,17^.

**Table 1:** Summary of Transgenic Analysis.

Fragment abbreviation	Precise Size of fragment tested (bp)	Total number of transgenic embryos^†^	Number with any limb expression	Number with ‘ZED-like’ limb staining^‡^	Cloning by: endogenous restriction site, retrieval or PCR^¶^	Genomic coordinates^***^
11.5 kb	11,514	N/D*	6	5	SnaBI	816,903–828,416
7.2 kb	7,232	6	1	0	SnaBI	828,467–835,698
22 kb	22,421	3	0	0	Retrieval	780,255–802,675
15 kb	15,028	3	0	0	Retrieval	802,264–817,291
5.2 kb	5,288	7	0	0	HindIII	816,903–822,190
6.2 kb	6,230	N/D	7	7	HindIII	822,187–828,416
2.9 kb	2,868	4	1	0	NruI	822,187–825,054
3.3 kb	3,362	N/D	8	8	NruI	825,055–828,416
1.3 kb	1,375	9	3	3	PCR	827,128–828,502
930 bp	930**	7	5	5	PCR	827,128–828,057
461 bp	461**	6	3	1^§^	PCR	828,042–828,502
563 bp	563	12	7	6	PCR	827,128–827,691
2,774 bp	2,774	5	5	5	PCR	827,128–829,901
CNE7	656	12	3	0	PCR	780,580–781,235

^†^As determined by PCR on DNA from yolk sacs.

^‡^Refers to staining that gave the same pattern in the limbs as the 18.8 kb transgene.

^¶^Subcloning was from the 18.8 kb βlacZ clone, using these restrictions sites and bordering sites from the multiple cloning site. The SnaBI digest removed 51 bp, but this sequence was retained in the 1,375 and 461 bp fragments.

^§^We scored this single embryo as “ZED-like” since dark staining covered the entire ZED domain (embryo at extreme lower right in Fig. 2E).

*Genotypes of all embryos were not determined (N/D) during intermediate screening steps when obvious ‘ZED-like’ staining was obtained in multiple embryos.

**The 930 and 461 bp PCR products had 16 bp of overlap.

***All coordinates are for Chromosome X, Genome version GRCh37/hg19.

### A 563 bp fragment is sufficient for ZED activity in a transgenic mouse assay

**Figure 2:**
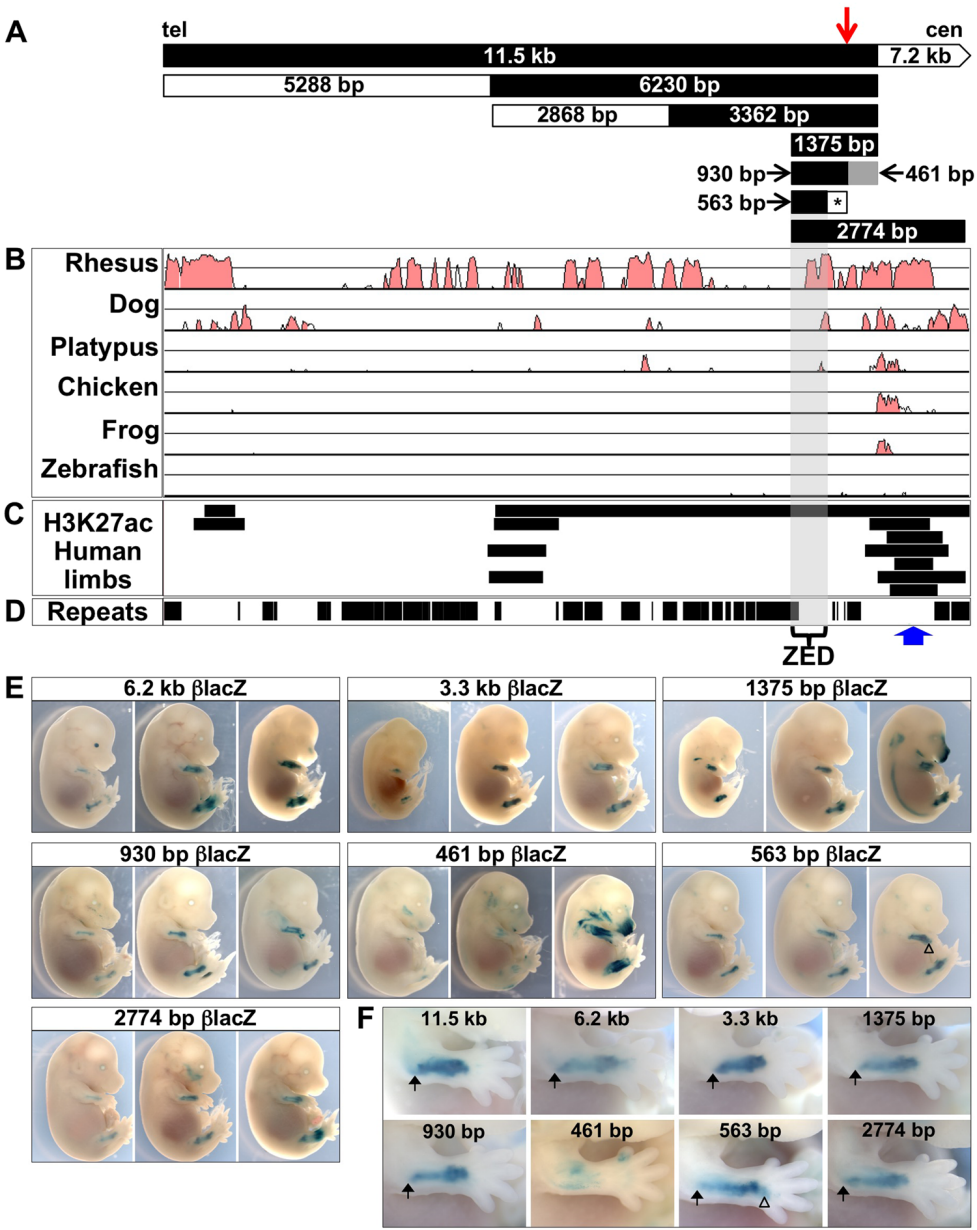
A 563 bp sequence is sufficient for ZED transgene activity. (**A**) Schematic map of nine fragments tested in the transgenic mouse assay, black fragments reproducibly tested positive for activity similar to that of the 11.5 kb βlacZ transgene. The 461 bp fragment is shaded grey to indicate its variable staining. The asterisk marks a 324 bp fragment that tested negative in a luciferase assay (Fig. 3C). (**B**) VISTA plot of evolutionary conservation among the indicated species aligned with the fragments from A. The 563 bp sequence with ZED activity is shaded and contains a small peak of conservation among non-rodent mammals (see Fig. 3). (**C**) Regions of H3K27ac in human embryonic limbs arranged as in Fig. 1B from Cotney *et al*.^29^. Note that the only region with enriched H3K27ac in all samples is within 1 kb (centromeric (cen)) of the 563 bp ZED and contains sequences conserved in mammals, chicken and frogs. (**D**) Repetitive elements from the UCSC genome browser. The blue arrow indicates the location of enriched H3K27ac in seven human limb samples^29^. (**E**) Sets of three E13.5/E14.5 embryos carrying the indicated transgenes. For each set, the embryo with the most staining obtained is shown at right, with a more moderately stained embryo in the middle and a weakly stained embryo at left. (**F**) Close-up of the forelimbs from each of the middle transgenic embryos from E, with fragment sizes indicated. The arrows show the location of the elbow where ZED staining is restricted to the posterior of the limb. The open triangles indicate area of gained expression in the autopod in the 563 bp βlacZ transgenics.

We subsequently subdivided the 11.5 kb sequence into progressively smaller fragments while tracking ZED activity with our transgenic embryo assay (Fig. 2). This strategy ultimately identified a 563 bp sequence that was sufficient to generate the ZED expression pattern in transgenic embryos (Fig. 2E,F). The genomic coordinates of this fragment are ChrX:827,128–827,691 in GRCh37/hg19, which is approximately 7 kb telomeric to CNE9 and 401 bp telomeric to the 47.5 kb deletion breakpoint. For all positive fragments, ZED transgene expression in the forelimb is characterized by a narrow posterior domain at the elbow (arrows in Fig. 2F) that becomes broader as it extends distally to the wrist. Hindlimb expression is similarly stereotypical for each of the positive fragments, but in this case extending more proximally into the stylopodal domain (femur) as well as the zeugopod. The complete set of embryos with reporter expression in limbs is shown in Supplementary Fig. S1. The 1,987 bp telomeric sub-fragment of the 3,362 bp βlacZ transgene was not tested in the transgenic assay because it mostly consisted of repetitive sequences (Fig. 2A,D) and we successfully predicted that the 1,375 bp fragment would contain the ZED activity.

At its endogenous location, the ZED is found directly adjacent to a region of high sequence conservation that is enriched for the activating acetylation of histone H3 on lysine 27 (H3K27ac) in human embryonic limbs^29^ (location indicated by the blue arrow in Figs 1B and 2D). Although this adjacent sequence did not have reproducible limb activity when tested as part of the 7.2 kb βlacZ transgene, we wondered if it might modulate ZED activity when combined with the active 563 bp enhancer sequence. Therefore, we created a 2,774 bp βlacZ transgenic construct that spanned the ZED and the more centromeric conserved region and tested it in our transgenic assay. The embryos carrying this “broader ZED” transgene had enhancer activity that was indistinguishable from the other ZED constructs, indicating that this additional sequence did not affect ZED activity in the context of a transgene (Fig. 2E,F and Supplementary Fig. S1). Nonetheless, we hypothesize that the H3K27ac/conserved sequence may participate in ZED function in its remote, endogenous chromosomal location (see discussion).

Another sequence at the telomeric extreme of the 47.5 kb deletion interval has been implicated as a potential limb enhancer based on its interaction with the *SHOX* promoter in a chromosome conformation capture assay (3 C) performed on samples from chick limb buds^17^. This element contains the CNE7 sequence that was negative for limb expression in chick electroporation assays^16^. We tested the human CNE7 sequence in our transgenic assay and found that among 12 transgenic E12.5 embryos, three had detectable limb activity (Supplementary Fig. S1). We chose E12.5 for CNE7 transgenic analysis since this corresponds to the chick stage (HH26) used in the 3 C assay^17^. Only one CNE7 transgenic embryo showed strong limb expression, and in that case, the expression was in the distal limb where *SHOX* is not expressed, and likely represents a position effect. In addition, the 22 kb transgene containing CNE7 did not show any limb enhancer activity at E14.5 (Fig. 1C). Taken together the data from our assays do not support a role for CNE7 in the control of *SHOX* expression in limbs.

### A primary limb bud cell luciferase assay identifies active ZED sequences and implicates putative HOX binding sites in its regulation

**Figure 3:**
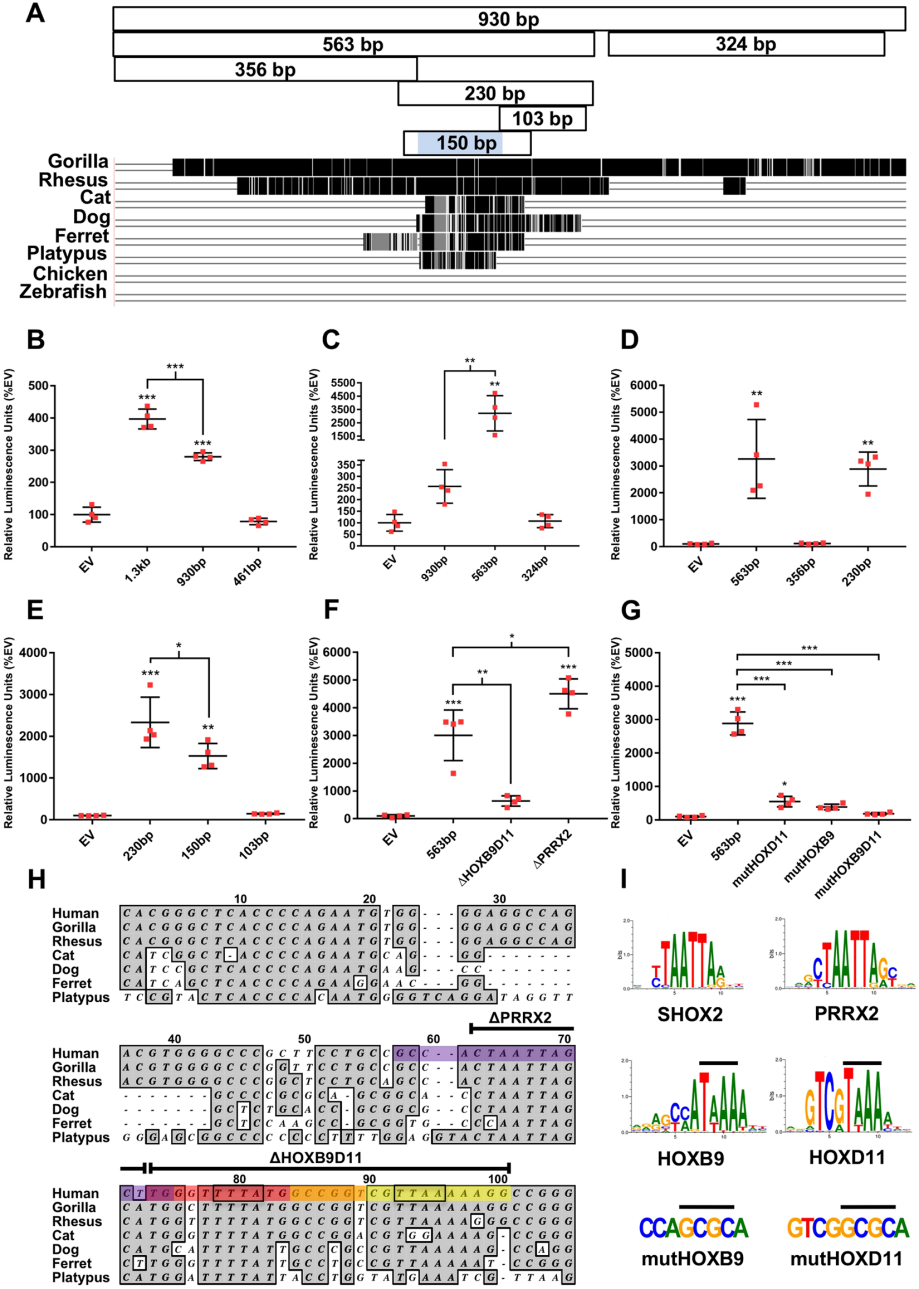
A Primary Limb Bud Cell Luciferase Assay further delineates ZED sequences with enhancer activity and identifies putative HOX binding sites required for ZED activity. (**A**) Map of sub-fragments of the 930 bp fragment tested with the luciferase assay, aligned above a track from the UCSC genome browser showing evolutionary conservation of the 930 bp in the indicated species. (**B**–**E**) Each panel represents a separate luciferase assay with the indicated fragments identified by their size. (**F**) Luciferase assay with the 563 bp ZED and the same fragment with a 28 bp deletion of the HOXB9/HOXD11 TFBSs (ΔHOXB9D11) or a 10 bp deletion of the core of two overlapping PRRX2 TFBSs (ΔPRRX2). The sequences deleted are shown in H. (**G**) Luciferase assay with the 563 bp ZED and the same fragment with site-specific mutations of the HOXD11 (mutHOXD11) or HOXB9 (mutHOXB9) TFBSs or mutations in both sites (mutHOXB9D11). The activity of each fragment was measured in duplicate limb bud samples from four E11.5 embryos with individual data points shown. Error bars show the SD. Statistical significance: *p < 0.03; **p < 0.003; ***p < 0.0001 in one-way ANOVA analysis with a Tukey’s post hoc test performed with GraphPad Prism. (**H**) Alignment of the conserved core sequence of the ZED in the indicated seven mammals (location of the 100 bp core shaded blue in A). The yellow and red shaded bases indicate the predicted HOXD11 and HOXB9 binding sites respectively, as identified by MatInspector, with orange representing the overlap of the two sites. The HOXB9 binding site is on the complementary strand. The core of two overlapping PRRX2 binding sites (one on each strand) is shown in purple. The sequences deleted in the constructs used in F are indicated. The bases mutagenized in the HOXB9 and HOXD11 sites are boxed. (**I**) The consensus binding sites for SHOX2, PRRX2, HOXB9 and HOXD11^36^. Images from Matbase (Genomatix). Lines above the HOXB9 and HOXD11 consensus sites indicate the bases that were replaced by mutagenesis. The mutagenized core sequences are shown below, with the changed bases indicated by lines.

In order to facilitate the more efficient screening of sequences for enhancer activity, we transfected luciferase reporter plasmids containing various candidate sequences into dissociated E11.5 mouse limb bud cells, adapting methods previously used for chick limb buds^30^. After transfection, the primary cells were cultured for 40 to 45 hours in micromass culture before measuring firefly and *Renilla* luciferase activity (the latter is for normalization, see Methods). We first validated this method by measuring the enhancer activity of the 1,375 bp (abbreviated 1.3 kb) and 930 bp fragments (Fig. 3B). Interestingly, the 930 bp fragment had significantly less activity than the 1,375 bp fragment in this assay, although the remaining sub-fragment (461 bp) did not have any measurable activity (Fig. 3B), perhaps indicating that this fragment contributes to the magnitude of ZED activity.

We then used the primary limb bud cell luciferase assay to analyze two sub-fragments of the 930 bp, which identified the 563 bp fragment as the active sequence (Fig. 3C). As noted above, our transgenic assay confirmed that this 563 bp fragment contains limb enhancer activity *in vivo* (Fig. 2E,F). Surprisingly, the 563 bp fragment was found to have markedly higher activity (approximately 10-fold) in the luciferase assay than the larger 930 bp sequence. We hypothesize that the remaining 367 bp contains repressive sequences that may be involved in fine tuning the expression domain of *SHOX*. This hypothesis is modestly supported by our transgenic results with the 563 bp enhancer, which consistently showed a slightly expanded expression domain into the autopod (open triangles, Fig. 2E,F). Note that the luciferase assay is performed on cells from earlier stage embryos (E11.5) than the E14.5 transgenic assay because micromass cultures are dependent on easily dissociated cells that have not yet terminally differentiated.

We further exploited the luciferase assay to delineate the active sequences within the 563 bp sequence (a map of the fragments tested is shown in Fig. 3A). These results support the conclusion that much of the enhancer activity is found within a 230 bp sub-fragment of the 563 bp (Fig. 3D). This 230 bp sequence contains a 100 bp interval that is well-conserved in non-rodent mammals, but not other tetrapods (Fig. 3A,H). A 150 bp fragment containing this conserved sequence also had high activity in the luciferase assay (Fig. 3E). We used the MatInspector software^31^ to identify candidate transcription factor binding sites (TFBSs) within the conserved 100 bp core. Among these were predicted binding sites for HOX9 and HOX11 proteins (red and yellow respectively in Fig. 3H; see discussion for a description of HOX9/11 proteins), as well as PRRX transcription factors (PRRX1/2) (purple, Fig. 3H). PRRX1/2 are paired-like homeodomain transcription factors that are closely related to SHOX and SHOX2^32^. These transcription factors all have prominent functions in stylopodal and/or zeugopodal limb development^33–35^.

We tested the importance of the predicted TFBSs for ZED activity by deleting and mutagenizing the specific sequences from the 563 bp ZED. We then measured the activity of the resulting constructs in primary cell luciferase assays (Fig. 3). Deletion of the 28 bp containing the overlapping HOX9/HOX11 TFBSs dramatically reduced the activity of the ZED by approximately 80% in the luciferase assay (Fig. 3F). Furthermore, we used site-directed mutagenesis to determine the importance of individual HOX TFBS for ZED activity. Mutagenizing the core four bp of either the HOX9 or HOX11 TFBS (from a TAAA or TTAA, respectively to GCGC), reduced the activity of the ZED to a level comparable to deletion of both sites (compare Fig. 3G and F), indicating synergistic function of these sequences in regulating the ZED in this assay. Mutagenesis of both HOX sites in the same construct reduced ZED activity even further (Fig. 3G).

Surprisingly, deletion of the 10 bp core sequence of two overlapping PRRX2 binding sites significantly increased ZED activity by approximately 50% (Fig. 3F), indicating that this site might function to inhibit ZED activity. The putative PRRX2 binding site identified by MatInspector is very similar to the consensus sequence for the SHOX2 TFBS (Fig. 3I) and almost identical to the PRRX1 site^36^. Furthermore, since the human SHOX homeodomain has an identical amino acid sequence to mouse and human SHOX2, these homeodomains are expected to bind the same TFBS. Therefore, this sequence could be part of negative feedback or fine tuning of ZED activity by SHOX, SHOX2 and/or PRRX1/2.

### Screening of LWD patients for mutations within the ZED region

At the point in our study when the ZED had been narrowed down to a 930 bp fragment, we began a screen to determine if inactivating mutations or deletions within this interval existed in suspected LWD patients for whom no causative mutation had been found. We screened for *SHOX* mutations and deletions in the region using commercial Multiplex ligation-dependent probe amplification (MLPA) assays (P018E1, F1 or G1, MRC Holland, The Netherlands), self-designed MLPA assays for the upstream region of *SHOX*^37^ and sequences flanking *SHOX* exon 6a^38^, and High resolution melting (HRM) and DNA sequencing of the *SHOXa* transcript (NM_000451.3). A 1026 bp fragment containing all but the initial 12 bp of the 930 bp fragment was PCR amplified from patient DNA and screened for mutations by Sanger sequencing. Eleven variants, nine single nucleotide variants (SNVs) and two single nucleotide insertions were identified in the cohort of 124 suspected LWD patients (Supplementary Table S2). Two of the SNVs, rs60406052 and rs142683771, present in this cohort were absent from the Spanish normal stature control cohort. However, all nine SNVs are now known to be present at different frequencies in some populations (gnomAD^39^, Supplementary Table S2) and are therefore unlikely to contribute to the LWD phenotype. Deletions and duplications in the region of interest were screened using a custom-designed MLPA assay in 104 of the 124 suspected LWD patients for whom sufficient DNA was available (Supplementary Table S3). No deletions or duplications were detected.

## Discussion

The majority of non-coding deletions linked to a *SHOX*-deficient phenotype remove the ZED^9,13,15,17,18,40^. Several of these, including the recurrent 47.5 kb deletion, leave the nearby CNE9 sequence intact. In contrast, to our knowledge no pathogenic deletions have been reported that remove CNE9 but leave the ZED intact. Therefore, we propose that the ZED is a more likely candidate than CNE9 to be a critical limb enhancer in the analyzed downstream region. In this view, CNE9 was implicated as a limb enhancer in part due to its proximity to the ZED. The ZED sequence was apparently overlooked in earlier studies because of its weak conservation; however, others have reported developmental enhancers with poor evolutionary conservation^41,42^. Our results demonstrate the utility of methodically analyzing a deletion interval for enhancer activity rather than assuming that an enhancer will be the most conserved sequence in a genomic region. Nonetheless, the other CNEs identified to date may be *SHOX* enhancers in other spatial or temporal contexts.

Importantly, the ZED is located in a position that could potentially explain the pathogenicity of the 47.5 kb recurrent deletion. Specifically, the centromeric breakpoint of the 47.5 kb deletion is located within the 461 bp fragment, 37 bp from the centromeric boundary of the 930 bp fragment such that it removes the enhancer from the genome (breakpoint shown by the red arrow, Fig. 2A). Since the 461 bp fragment displayed some limb enhancer activity in our transgenic assay, its retention after deletion of the 47.5 kb fragment could potentially support some degree of limb expression. Studies of families with this 47.5 kb deletion have shown that while some individuals may have a few or all of the LWD clinical characteristics, other family members with the deletion have normal limbs^17,18^. Thus, this deletion appears to have an incomplete or reduced penetrance. Despite the identification of a novel limb-specific enhancer within this deleted sequence, we still cannot explain the observed incomplete penetrance and the variable clinical heterogeneity. However, even mutations removing or mutating *SHOX* coding sequences have remarkably variable penetrance. This issue has recently been addressed by Montalbano *et al*. who reported a *SHOX* modifier, retinoic acid catabolising enzyme CYP26C1^43^. *CYP26C1* mutations were identified in three families in which pathogenic *SHOX* mutations were also present. Only the individuals with both mutations presented with the classical LWD phenotype. However, the frequency of mutations in this modifier was very low with only two cases being identified in a cohort of 68 LWD individuals. The estimated frequency of having a mutation in both *SHOX* and *CYP26C1* was 1.8 × 10^−5^. Thus, other modifiers remain to be identified.

A core ~100 bp of the ZED sequence is conserved in mammals, with the exception of rodents (Fig. 3A,H). The identification of a relatively short sequence responsible for ZED activity could serve as a gateway for identifying how *SHOX* is integrated into the gene regulatory networks that control limb patterning, especially since the ZED contains conserved, putative TFBSs for the HOXB9 and HOXD11 proteins (Fig. 3). Since HOXB9 does not function in patterning the proximal-distal axis of the limb, it is important to note that the identified HOXB9 TFBS is almost identical to that of the paralogous HOXA9/D9 proteins^36,44^ (collectively referred to as the HOX9 proteins) that are expressed in the developing zeugopod and have an important function in proximal limb development^45^. In addition, HOXA9 has previously been implicated in the regulation of *SHOX*^46^. Similarly, the predicted HOXD11 TFBS is identical to that of its paralog HOXA11, as determined by protein-binding microarrays^36^. The *HOXA11/HOXD11* genes are particularly intriguing candidates as potential upstream regulators of *SHOX* since their mutation in mice causes zeugopodal truncations similar to that of Langer mesomelic dysplasia patients^47^. Significantly, our results show that predicted HOX9 and HOX11 TFBSs are each required for the majority of ZED activity in a luciferase assay. Therefore, the ZED sequence could be an important tool for deciphering how *SHOX* fits into the HOX limb patterning system. In future experiments beyond the scope of the present study, a transgenic assay will be used to determine whether HOX9 and HOX11 proteins are required for ZED enhancer activity *in vivo*.

Analysis of epigenetic chromatin modifications has been another fruitful strategy for determining the genomic location of enhancers^48^. As noted above, the wild-type mouse genome cannot be used for this study since it lacks *SHOX*. However, Cotney *et al*. reported the genome-wide sites of H3K27ac, which labels active promoters and enhancers, in human embryonic limbs^29^. The regions showing enriched H3K27ac within the *SHOX* enhancer deletion interval are shown for limb tissue samples from E33, E41, E44 and E47 in the lower track in Fig. 1B. Interestingly, the most highly conserved sequences within the interval are either completely negative for H3K27ac enrichment (CNE7) or positive in only one of the seven samples (CNE8/CNE9), further suggesting that these sequences may not be limb enhancers. In contrast, the only sequence positive in all seven samples is within 1 kb of the ZED sequence (Blue arrow, Figs 1B and 2C). We did not detect any change in ZED activity when the more highly conserved/H3K27ac sequences were included in a transgene construct (2,744 bp βlacZ, Fig. 2E,F). However, the epigenetic modification of these neighboring regions could perhaps contribute to ZED function in its endogenous chromosomal context if, as we hypothesize, the enhancer must act at a distance to contact and activate the *SHOX* gene in developing limbs. The ZRS has been shown to contain sequences necessary for spatial activation of gene expression, such as those revealed by transgene assays, and other adjacent, yet distinct sequences required for long-range function^49^. Therefore, the ZED, like the ZRS, may have adjacent sequences that are required for function from its endogenous location. Ultimately, elucidating the definitive function of the ZED and neighboring sequences will likely require genomic targeting experiments in a non-rodent mammalian model whose genome contains both *SHOX* and the ZED. Furthermore, non-mammalian vertebrate genomes could be tested for functional ZED orthologs that have evaded detection because of a lack of obvious sequence conservation.

Although the ZED’s location and specific activity make it a promising candidate as a discrete *SHOX* limb enhancer, several reports of copy number variations (CNVs) in LWD and ISS patients implicate other genomic regions in the *cis*-regulation of *SHOX*. Three ISS families/patients have been reported with non-coding deletions upstream of *SHOX*^37,50^ and another study reported a homozygous >500 kb downstream deletion in members of a consanguineous family with LWD^51^. This latter deletion begins ~100 kb further downstream of *SHOX* than the ZED. Several studies have also linked duplications of PAR1 regions to LWD and ISS^50,52–54^. In some cases, these duplications include the *SHOX* gene itself^52,54^, whereas several affect only upstream or downstream non-coding regions, some of which include the ZED^53^. These duplications may interfere with the regulatory interactions necessary for *SHOX* expression, perhaps including perturbation of ZED function. Chromosomal interactions are now known to be confined mostly to discrete ~1 Mb regions called topologically associating domains (TADs)^55^. The disruption of TAD boundaries has been shown to alter interactions of limb enhancers with their target genes resulting in limb abnormalities in mice and humans^8^. *SHOX* is near the center of a TAD that consists of the most telomeric ~1.2 Mb of the X and Y chromosomes, with a boundary just telomeric to the *CRLF2* gene as determined in a variety of cell types^56,57^. Several CNVs disrupt the boundary of this TAD^50^, which may cause misregulation of the *SHOX* gene resulting in LWD and ISS in these patients. Our screen of 124 suspected LWD patients without a known causative mutation did not reveal any deletions or SNVs of the minimal ZED region that are not found in the unaffected, general population. This suggests that the ~47.5 kb deletion may be the smallest, common pathogenic deletion that removes the ZED. Taken together, these results highlight a need for further studies of the components and extent of the *cis*-regulatory landscape of the *SHOX* gene in order to understand the functional significance of CNVs in this genomic region.

## Methods

### Cloning of transgene constructs

All genomic fragments tested in transgenic constructs were cloned into the pβlacZ plasmid^26^, either as restriction fragments of the 18.8 kb βlacZ transgene, PCR products amplified from the same transgene, or by retrieval from bacterial artificial chromosomes (BACs) by gap repair as indicated in Table 1 and as described^26^. The primers used for cloning are listed in Supplementary Table S1. The 22 kb and 15 kb fragments were cloned by retrieval directly into pβlacZ from BACs RP13-76L22 and RP13-167H21, respectively. BACs were obtained from the Wellcome Trust Sanger Institute. CNE7 was generated by PCR from BAC RP13-76L22. The CNE7 PCR fragment used was identical to the ECR1 fragment from Benito-Sanz *et al*.^17^, which includes all of the CNE7 sequence analyzed by Sabherwal *et al*. as well as 135 bp of flanking sequence. All fragments generated by PCR were confirmed by sequencing. Retrieved fragments were confirmed by sequencing at each end and restriction enzyme analysis.

### Transgenic mice

All experiments using mice were performed in accordance with Canadian Council on Animal Care guidelines as approved by the University of Calgary Life and Environmental Sciences Animal Care Committee, Protocol # AC13-0053. Transgenic embryos and permanent lines were produced by pronuclear injection of DNA constructs into CD-1 one-cell stage embryos using standard techniques^58^. All transient transgenic embryos and lines were produced at the University of Calgary Centre for Mouse Genomics. Mice were genotyped by forward primers specific for each transgene and a common reverse primer within the lacZ sequence. Transgenic embryos were stained with X-gal using standard techniques^58^.

### Patient and control cohorts

A total of 124 patients with suspected LWD, without alterations in *SHOX* or its known enhancers were included in this study which was performed in accordance with the tenets of the Declaration of Helsinki. The inclusion criteria included the presence of short stature and/or mesomelic shortening of the limbs and/or bilateral Madelung deformity in the proband or a direct family member. All participants provided informed consent for the performed studies and ethical approval was obtained from Hospital La Paz, Madrid (PI-1387, PI-2630). Genomic DNA was isolated from peripheral blood using the Blood kit (QIAGEN, Valencia, CA) or Chemagic DNA extraction special kit (Chemagen, Perkin Elmer Inc, Germany). A cohort of 126 healthy controls with heights within the normal range for the Spanish population for age and gender were obtained from the Spanish DNA bank (University of Salamanca, Salamanca, Spain).

### Mutation Sequence Analysis

The screening of a 1026 bp fragment that included all but the most telomeric 12 bp of the 930 bp fragment with expression in the limbs of transgenic embryos was performed using direct sequencing. The slight difference in fragment size was due to the difficulties in primer design for amplifying sequences from human genomic DNA. The oligonucleotides for PCR amplification were RCE-P4_F1 5′-ATGGACACACAACACAGTTT-3′ and RCENP4_R1 5′-AATCGTGACCATCATACTC-3′. Amplified products were directly sequenced with the amplification primers and four internal sequence primers RCE-P4_SeqF1 5′-ACTTTAAGTTTTAGGGTACAGGTG-3′, RCE-P4_SeqR1 5′-GGCGTCCTCATGGGGA-3′, RCE-P4_SeqF2 5′-CCGAGACGTCGTCACGA-3′ and RCE-P4_SeqF3 5′-GGGTTTGCAGCAAAGTG-3′. The PCR product was subsequently sequenced using the BigDye Terminator V3.1 kit (Life Technologies, Thermo Scientific Inc) and electrophoresed on an ABI3730XL sequencer (Applied Biosystems).

Population frequencies of the detected variants were assessed by screening 126 Spanish normal height controls and using the Genome Aggregation Database (gnomAD browser beta, http://gnomad.broadinstitute.org/) or dbSNP (https://www.ncbi.nlm.nih.gov/).

### PAR1 deletion/duplication detection

Screening for the presence of deletions and duplications within the 930 bp region in the *SHOX* downstream region was undertaken using a custom designed Multiplex ligation-dependent probe amplification (MLPA) which encompassed ~11 kb (ChrX:817,716 to 828,838 in GRCh37/hg19, Supplementary Table 3) and analyzed as previously described^17^.

### Primary Cell Luciferase Assay

Each biological replicate for the luciferase assay consisted of cells isolated from fore- and hindlimb buds dissected from a single CD-1 E11.5 embryo as described^59^, with minor modifications using n = 4 littermates per condition. Limb buds were incubated in 0.8 units/ml dispase (Gibco) for 1.5 hours at room temperature, followed by 0.25% trypsin for 20 minutes at 37 °C. Single-cell suspensions were then created by repeated pipetting of the limb buds in micromass media (3:2 F12:DMEM, 10%FBS, 100 µg/ml Normocin) and diluted to approximately 1 × 10^7^ cells/ml for each animal. Cells from 20 µl of each suspension were transfected as described^59^ using 1 µg of reporter plasmid (the enhancer construct as cloned into pGL4.23 *luc2/*minP plasmid (Promega)) together with 5 ng pRL-TK *Renilla* (Promega). 10 µL micromass cultures (~1 × 10^5^ cells) were spotted in duplicate for each animal and allowed to adhere for 60–70 minutes at 37 °C, 5% CO_2_, before feeding with 600 µL media and incubating an additional ~40–45 hours. Firefly luciferase luminescence was read for each well ~1 hr after addition of Dual-Glo luciferase reagent (Promega), then incubated a further 20–30 minutes after adding Dual-Glo Stop & Glo reagent before reading *Renilla* luciferase luminescence. The ratios of firefly to *Renilla* luminescence values were calculated for each well, and averaged between the duplicate cultures. The means (+/−standard deviation (SD)) of four of these normalized values (biological replicates) were calculated and presented as a percentage of the values obtained for empty vector (EV) transfections. GraphPad Prism was used for statistical analysis using a one-way ANOVA with Tukey’s post hoc test for multiple comparisons.

### Deletions and site-directed mutagenesis within ZED luciferase constructs

All deletions and mutations were introduced into the 563 bp ZED fragment. Deletions were created with PCR SOEing^60,61^ using the primers in Supplementary Table S1 and the 563 bp fragment as a template. For each deletion two PCR products were synthesized with Precision Taq (abm, Inc.). The products were mixed and a filling reaction without primers was performed as described^61^, followed by the SOEing reaction using the forward primer from Product 1 and the reverse primer from Product 2. The resulting fragment was cloned into pGL4.23 *luc2/*minP after Acc65I/SalI digestion. In order to simultaneously remove predicted HOXB9 and HOXD11 binding sites, 28 bp were deleted as shown in Fig. 3H to produce construct ΔHOXB9D11. Similarly, the core of two predicted and overlapping PRRX2 binding sites were deleted by removing 10 bp (Fig. 3H) resulting in construct ΔPRRX2.

Mutations of specific TFBSs were performed on the 563 bp ZED-pGL4.23 *luc2/*minP plasmid with the primers in Supplementary Table S1 using the QuikChange II site-directed mutagenesis kit (Agilent) according to the manufacturer’s instructions. In each case a core sequence of TAAA (HOXB9) or TTAA (HOXD11) (boxed sequences in Fig. 3H) was changed to GCGC as described^62^ (Fig. 3I), to produce the mutHOXB9 and mutHOXD11 site. A second reaction was performed to mutagenize the HOXD11 binding site within the mutHOXB9 construct resulting in the double-mutant mutHOXB9D11 plasmid. All deletions and mutations were confirmed by sequencing and the verified constructs were used in the primary cell luciferase assay as described above.

### Sequence analysis

Genomic sequences from all species were retrieved from the UCSC genome browser and uploaded to the VISTA website where they were aligned with MLAGAN and analyzed for conservation with mVISTA^63,64^. All human sequences and tracks were from genome version GRCh37/h19. Candidate transcription factor binding sites were identified with MatInspector from Genomatix^31^.

## Electronic supplementary material

Supplementary Information
