# Supplementary material for: Identification of a limb enhancer that is removed by pathogenic deletions downstream of the SHOX gene
Source: Sci Rep. 2018 Sep 24;8:14292. doi: 10.1038/s41598-018-32565-1 (PMC6155277; doi:10.1038/s41598-018-32565-1)
Supplement: Supplementary file 1 — Supplementary Information [file 41598_2018_32565_MOESM1_ESM.pdf]

Supplementary Information for:
Identification of a limb enhancer that is removed by pathogenic deletions downstream of the *SHOX* gene

Isabella Skuplik[1], Sara Benito-Sanz[2], Jessica M. Rosin[1] Brent E. Bobick[1], Karen E. Heath[2]* and John Cobb[1]*

[1]Department of Biological Sciences, University of Calgary, 2500 University Drive N.W., Calgary, Alberta T2N 1N4, Canada

[2]Instituto de Genética Médica y Molecular (INGEMM), IdiPAZ and Skeletal dysplasia multidisciplinary unit (UMDE), Hospital Universitario La Paz, Universidad Autónoma de Madrid, Pº Castellana 261, 28046 Madrid, Spain and CIBERER, ISCIII, Madrid, Spain

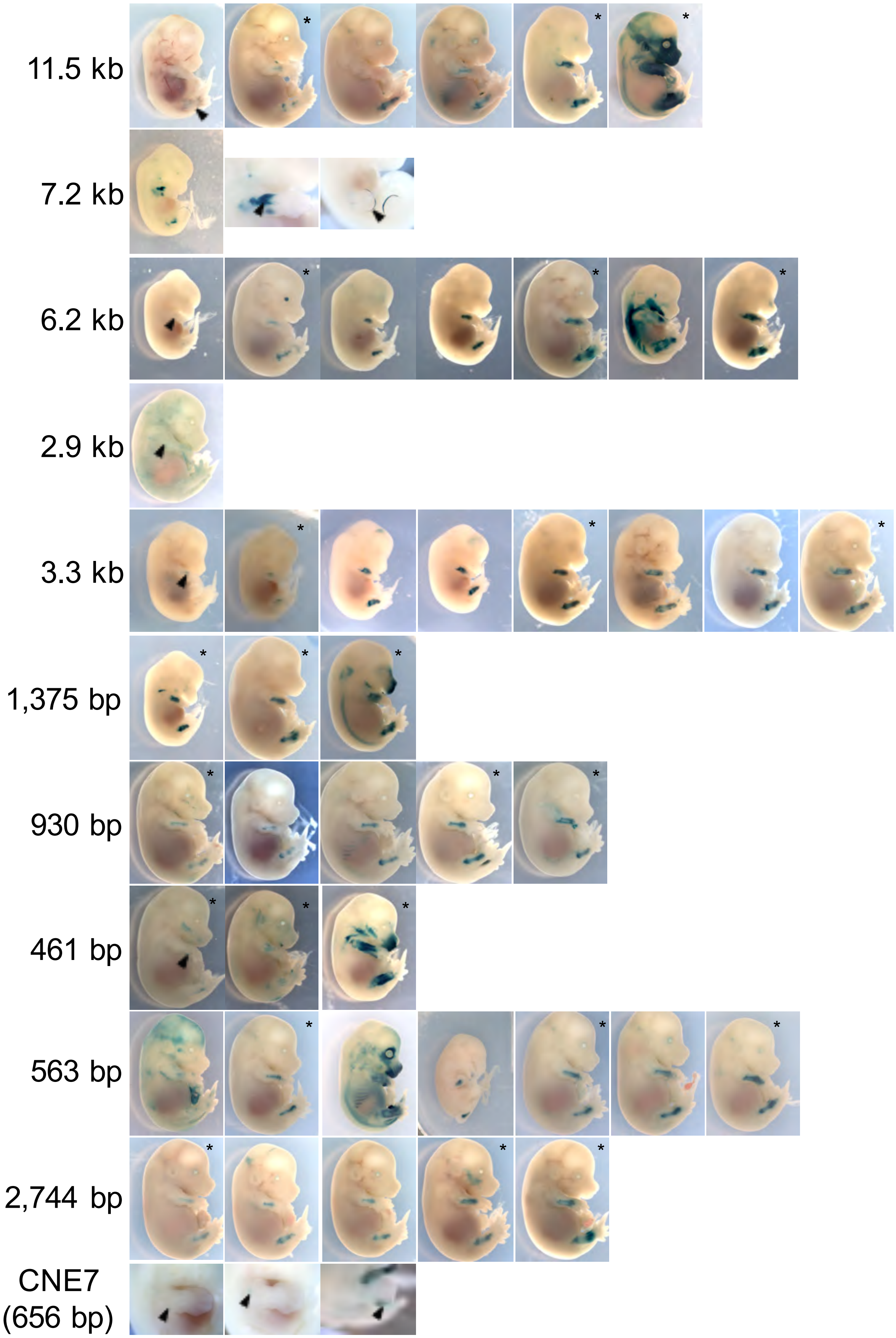

11.5 kb
7.2 kb
6.2 kb
2.9 kb
3.3 kb
1,375 bp
930 bp
461 bp
563 bp
2,744 bp
CNE7
(656 bp)

Supplementary Figure S1. **The complete set of transgenic embryos positive for limb staining.** Transgenic constructs are indicated by fragment size at left. Embryos are arranged from least to most intensely stained in limbs. The asterisks indicate those embryos used in Fig. 1 or 2. Arrowheads point to areas of weak limb staining. All embryos are transient transgenics, with the exception of the 7.2 kb fragment for which permanent lines were created. The three images shown for the 7.2 kb fragment are all from the single line that showed limb staining (5 other lines were negative for limb staining). The close-up of an E13.5 limb (middle image) from the 7.2 kb line shows that the staining is limited to the periphery of the limb (arrowhead) and does not stain the interior mesenchyme as seen for the ZED fragments. At E11.5 (image at right), this 7.2 kb fragment transgene has activity in the apical ectodermal ridge, which was not scored in any of the 18.8 kb transgenic lines. Therefore, this staining appears to be a position effect.

For the CNE7 fragment, close up views of the limbs are shown: two of twelve E12.5 transient transgenic embryos showed very weak staining in the proximal limb (arrowheads) and a third embryo (at right) showed distal limb expression. The ZED also has strong limb activity at E12.5 [23].

Supplementary Table S1

PCR primers* used for cloning fragments for transgenic and luciferase constructs

1,375 bp	F: TAA*ggtacC*TCAAGACACACATGGACACACAAC
	R: AATA*gtcgac*AATTGCCATTCCCGCAGTGC
930 bp	F: TAA*ggtacC*TCAAGACACACATGGACACACAAC
	R: AATA*gtcgaC*ACCAGCATGGCACATGGATAC
461 bp	F: TAA*ggtacc*ATGTGCCATGCTGGTGTGGTGC
	R: AATA*gtcgac*AATTGCCATTCCCGCAGTGC
563 bp	F: TAA*ggtacC*TCAAGACACACATGGACACACAAC
	R: AATA*gtcgac*AGGACGCCAAAGTTCAGGAGA
2,774 bp	F: TAA*ggtacC*TCAAGACACACATGGACACACAAC
	R: AATA*gtcgac*TCCAGAATATGTGAATGGCACG
CNE7	F: TAA*ggtaCC*CTTGTCCATCTGCGTCTAC
	R: AATA*gtcgac*CTAACGGCTCACATGAAACTG
324 bp	F: TAA*ggtaCC*TCATGGGGAAAAAAAAATGTAA
	R: AATA*gtcgac*TGCACCTGTACCCTAAAACTTAAAGTATAA
356 bp	F: TAA*ggtacC*TCAAGACACACATGGACACACAAC
	R: AATA*ctcgag*GACTCCTGCTCACACTTTGCTG
230 bp	F: TAA*ggtacc*GCAGCAAAGTGTGAGCAGGAG
	R: AATA*ctcgag*AGGACGCCAAAGTTCAGGAGA
150 bp	F: TAA*ggtacc*GCAGCAAAGTGTGAGCAGGAG
	R: AATA*ctcgaG*CTGTTTCATAACTTTGCAACTG
103 bp	F: TAA*ggtacC*AGTTGCAAAGTTATGAAACAGCC
	R: AATA*ctcgag*AGGACGCCAAAGTTCAGGAGA

PCR Primers for 22 kb fragment retrieval by gap repair

22kb 369 bp 5' homology arm
F: GAGGTTTCACTAT*GGTACC*CAGGTT
R: GTC*agatct*AGGATGATGAGTAGACGCAGATGG

22kb 412 bp 3' homology arm
F:GTC*agatct*AGACCCTAACTGATCCAGCAGC
R: TCTG*gtcgac*GCACACTCACACAAACACAGGG

PCR Primers for 15 kb fragment retrieval by gap repair

15kb 412 bp 5' homology arm
F: ATAA*ggtacc*AGACCCTAACTGATCCAGCAGC
R: GTC*agatct*GCACACTCACACAAACACAGGG

15kb 389 bp 3' homology arm
F: GTC*agatct*GGAACAGAAAACCAAACACCGC
R: TCTG*gtcgac*GAATAACTGCCTGGATGTGACAGC

Primers for PCR SOEing (The transition from blue to black sequence represents the point of fusion of the deletion)

Deletion of the HOXB9 and HOXD11 binding sites from the 563 bp ZED fragment

Product 1
F: TAA*ggtacc*TCAAGACACACATGGACACACAAC
R: CAACTGTTTGACCCGGAGCTAATTAGTGGCGGCAGG

Product 2
F: CCGCCACTAATTAGCTCCGGGTCAAACAGTTGCAAA 3’
R: AATA*gtcgac*AGGACGCCAAAGTTCAGGAGA 3’

Deletion of the PRRX2 core binding site from the 563 bp ZED fragment

Product 1
F: TAA*ggtacc*TCAAGACACACATGGACACACAAC
R: CCGGCCATAAAACCCATGGCGGCAGGAAGCGG

Product 2
F: CCGCTTCCTGCCGCCATGGGTTTTATGGCCGGTC
R: AATA*gtcgac*AGGACGCCAAAGTTCAGGAGA

Supplementary Table S1

**Site-directed Mutagenesis primers**
Sequence in red indicate the base changes introduced during mutagenesis
Primers for mutation of the HOXB9 binding site
F: GCTTCCTGCCGCCACTAATTAGCTTGGGTGCGCTGGCCGGTCGTTAAA
R: TTTAACGACCGGCCAGCGCACCCAAGCTAATTAGTGGCGGCAGGAAGC
Primers for mutation of the HOXD11 binding site
F: CTTGGGTTTTATGGCCGGTCGGCGCAAAGGCCGGGTCAAACAGTTG
R: CAACTGTTTGACCCGGCCTTTGCGCCGACCGGCCATAAAACCCAAG
Primers for mutation of the HOXD11 binding site after HOXB9 mutagenesis
F: TGTTTGACCCGGCCTTTGCGCCGACCGGCCAGCGCACC
R: GGTGCGCTGGCCGGTCGGCGCAAAGGCCGGGTCAAACA

*Sequences in red and italicized are restriction sites used in cloning (either Acc65I, SalI, XhoI or BglII). Sequence 5' to these is to facilitate cutting.

Supplementary Table S2: Allelic frequency of variants detected within fragment 6 (930 bp) in the suspected LWD cohort and Spanish normal height control cohort and those reported in the different populations in dbSNP or gnomAD. The genomic coordinates are according to chromosome X genomic sequence, NC_000024.10 (GRCh37/hg19). Two of the SNVs were absent from the control cohort (indicated in bold). Abbreviations: AFR, African/African American; ASJ, Ashkenazi Jewish; EA, East Asian; FIN, Finish European; EUR, European (Non-Finnish); AMR, Latino.

		**LWD cohort**		**Control Cohort**		**Variant allele frequency**						
**dbSNP ID**	**NC_000024.10**	**Number of alleles**	**Variant allele frequency**	**Number of alleles**	**Variant allele frequency**	**AFR**	**ASJ**	**EA**	**FIN**	**EUR**	**AMR**	**Other**
rs5946518	g.827325 G>A	248	0.2823	252	0.3214	0.3836	0.2637	0.4081	0.2777	0.3087	0.4239	0.2990
rs5946519	g.827397 G>C	248	0.1452	252	0.1270	0.1125	0.1130	0.0169	0.0959	0.1253	0.1522	0.1088
rs5946520	g.827550 T>G	248	0.4194	252	0.4048	0.5683	0.3483	0.3176	0.3176	0.4118	0.4231	0.3832
rs73180471	g.827681 T>A	248	0.0806	252	0.0397	0.0240	0.0479	0.0406	0.0240	0.0694	0.1076	0.0578
rs201997827	g.827710_827711insA	186	0.4247	230	0.4609	0.2050	-	0.4603	-	0.466	-	-
rs68084433	g.827790 A>T	208	0.4952	228	0.5044	0.5151	0.4208	0.4180	0.1847	0.4745	0.3917	0.4332
rs111882096	g.827940_827941insG	208	0.9519	217	0.9816	0.9991	-	1.000	-	0.9661	-	-
**rs142683771**	**g.828069 T>G**	**248**	**0.0323**	**252**	**0.0000**	0.0051	0.0034	0.0006	0.0077	0.0332	0.0105	0.0226
rs187888356	g.828093 G>A	248	0.0081	252	0.0079	0.0020	0.0034	0.0125	0.0026	0.0031	0.0105	0.0030
rs28756673	g.828102 G>A	248	0.0081	252	0.0079	0.1414	0.0105	0.0000	0.0025	0.0032	0.0092	0.0039
**rs60406052**	**g.828119 G>C**	**248**	**0.0081**	**252**	**0.0000**	0.1608	0.0041	0.0000	0.0789	0.0030	0.0710	0.0173

Supplementary Table S3: Custom designed MLPA utilized to screen deletions and duplications in a 3.1 kb region encompassing the ZED in PAR1. The MLPA probes are listed in order from the telomere to centromere of the chromosomes X/Y, extending from X:817716 to 828838 (GRCh37/hg19). The probes P07, P09, P10, P11, P12 and the three autosomal control probes (*WBSC1, RIT2* and *RNF125*) are as previously described by Benito-Sanz et al. [12]. Five new probes were designed between P11 and P12, (X:825239-828790) and two of these, P11.3 and P11.4 are located within the 563 bp ZED. The ligation site is located between the 3´ end of the left probe and the 5´ end of the right probe. Right probes are 5´- phosphorylated.

Probe name	Locus	Localization (GRCh37/hg19)	Left probe sequence (5´>3´)	Right probe sequence (5´>3´)	Size (bp)
P07	PAR1	X:817716-817779	CGATGTTCTGTAAGTTGCATGTCTGCT	TTTTGAATGGCCCCTGTGGTTTTTGGAGTTCCTCTCCCATG	125
P09	PAR1	X:822083-822130	CTTTGCTCGGAACGTAGAGGA	AAAGACGTCCCTCCTGCTCCGGGAATC	110
P10	PAR1	X:823169-823238	CTGCAATTTGGGTCTCTCAGAAACCTCATT	TTAACAAACCGCATTCTCCAATTCCCTCTCTCTGTGTGCGCATG	118
P11	PAR1	X:825189-825239	GGTTCATCAAATACCATGCGGTGCTT	TTGCAGGTGGAAAGCTGCAGGATTG	131
P11.1	PAR1	X:825703-825759	GCCAATACCAATCTTGCTTCAACCCACT	TTCCCCCTTGGTTGGTCCGAGGTGCAAAC	99
P11.2	PAR1	X:826393-826446	CCACACATGGGATCAACAGACACTA	ATACGCAACACACATTCAACAGACAAATG	96
P11.3	PAR1	X:827322-827374	GCAGCGTCCATCATGGGGACCTTTGT	TTGCTTCCCCCTCTATGGGAAAATTAC	107
P11.4	PAR1	X:827628-827678	CTGGGCTTTGCAGAGGTATGAATGT	TTCGCTGTGTTGCCAACTTCTCCTG	92
P11.5	PAR1	828233-828280	GGTGCTTCTTGGGCCAGCTAGGAGATGA	TTGGGTTGTAAACTTGGGGGCTGGCTC	103
P12	PAR1	X:828790-828838	CGTCTACATCTTGGGCATGGCAGT	TTGGGCACCTATTTCAAGGGTGTGG	128
WBSC1	7q11.23	7:73604582-73604629	GGTAGCTCTCGAGAATCTAGAGGTGG	ATGGGATTCCCGGGATGACTTC	89
RIT2	18q12.3	18:40554039-40554110	GATGCTTATAAGACCCAGGTCAGGATTGACAATGAGCCAG	CTTACTTGGACATCTTGGACACTGCTGGCCAG	140
RNF125	18q12.1	18:29598506-29598564	GTCCTCTTCTCTGCAGTTGAGGTTTCAGGT	TTCAATCCTCCCAATACCACAAGACAGAG	115
